# Prognostic implication of intratumoral metabolic heterogeneity in invasive ductal carcinoma of the breast

**DOI:** 10.1186/1471-2407-14-585

**Published:** 2014-08-12

**Authors:** Seung Hyun Son, Do-Hoon Kim, Chae Moon Hong, Choon-Young Kim, Shin Young Jeong, Sang-Woo Lee, Jaetae Lee, Byeong-Cheol Ahn

**Affiliations:** Department of Nuclear Medicine, Kyungpook National University School of Medicine and Hospital, 50 Samduk-dong 2-ga, Jung-gu, Daegu, 700-721 Republic of Korea

**Keywords:** Intratumoral metabolic heterogeneity, Heterogeneity, Invasive ductal carcinoma, Breast cancer, ^18^F-FDG PET/CT, Prognosis

## Abstract

**Background:**

The purpose of this study was to evaluate the prognostic implication of findings of intratumoral metabolic heterogeneity on pretreatment ^18^F-FDG PET/CT scans in patients with invasive ductal carcinoma (IDC) of the breast.

**Methods:**

One hundred and twenty-three female IDC patients who underwent pretreatment ^18^F-fluorodeoxyglucose positron-emission tomography/computed tomography (^18^F-FDG PET/CT) scans were retrospectively evaluated in this study. The heterogeneity factor (HF) defined as the derivative (dV/dT) of a volume threshold function from 40% to 80%, was computed for each primary tumor. Other metabolic PET parameters (maximum standardized uptake value [SUVmax], metabolic tumor volume [MTV], and total lesion glycolysis [TLG]) were measured. The HF was compared with clinicopathologic factors and other PET parameters. Univariate and multivariate analyses for the overall survival (OS) were performed.

**Results:**

The HF ranged from 0.02 to 6.72 (mean, 0.35 ± 0.82) and significantly correlated with MTV (r = 0.955; p < 0.0001) and TLG (r = 0.354; p = 0.0001). The HF was significantly higher (implying more heterogeneity) in tumors with higher T and N stages. The optimal cut-off values for the OS determined using a receiver operating characteristic (ROC) curve were 0.34 for the HF, 5.6 for SUVmax, 8.55 cm^3^ for MTV, and 14.43 for TLG. The OS rate among the 123 patients was 86.2%. T stage (1, 2 vs. 3, 4), N stage (0, 1 vs. 2, 3), M stage (0 vs. 1), ER status (+ vs. –), SUVmax (≤ 5.6 vs. > 5.6), MTV (≤ 8.55 cm^3^ vs. > 8.55 cm^3^), TLG (≤ 14.43 vs. > 14.43), and HF (< 0.34 vs. ≥ 0.34) affected the OS on univariate analysis. After adjustment for the effects of TNM stage and ER status, the HF and MTV were significant predictors of OS. Among the PET parameters, the best prognostic factor for OS was the HF.

**Conclusions:**

Intratumoral metabolic heterogeneity correlated closely with the MTV and significantly affected the OS in IDC patients. The HF may act as a robust surrogate marker for the prediction of OS in IDC patients.

## Background

An important feature of malignant tumors is the heterogeneity of constituent cells and matrix caused by haphazard cellular proliferation, necrosis, and accumulation of extracellular material. Disorganized neovascularity in the tumor contributes to the heterogeneity. These heterogeneities appear as alterations in phenotypic features such as cellular morphological characteristics, gene expression, metabolism, and motility, and as variations in behavioral characteristics such as angiogenesis and changes in proliferative, immunogenic, and metastatic potential [1–6]. It is well known that both malignant and benign components make up most solid tumors [7–9].

Intratumoral metabolic heterogeneity has been well demonstrated by heterogeneous ^18^F-fluorodeoxyglucose (^18^F-FDG) distribution within tumors on autoradiography (ARG) [7]. There have been few reports regarding the biological mechanisms involved in the intratumoral heterogeneous distribution of ^18^F-FDG in tumors. Zhao *et al.* illustrated intratumoral distribution of ^18^F-FDG using ARG and compared it with regional expression of glucose transporter-1 (GLUT-1), glucose transporter-3 (GLUT-3), and hexokinase-II (HK-II) in a rat tumor model [10]. The study showed relatively high ^18^F-FDG accumulation in the central regions of the tumors. The uptake correlated with increased expression of GLUT-1, GLUT-3, and HK-II. Positive staining of hypoxia-inducible factor-1α (HIF-1α) was also observed in these regions. The results of that study suggest that intratumoral ^18^F-FDG distribution significantly correlates with the levels of GLUT-1, GLUT-3, and HK-II, which may stem from hypoxia within the tissues. Xu *et al.* more recently reported significant intratumoral heterogeneity of the mitochondrial redox state observed in xenografts of tumor tissues from breast cancer patients in nude mice [11]. They showed a correlation between the distribution of tumoral glucose uptake and the redox indices.

Although the causes of heterogeneous distribution of ^18^F-FDG within a tumor are not fully understood, several previous studies have demonstrated that heterogeneous ^18^F-FDG uptake is related to the heterogeneity of histopathologic features in various malignancies, including non–small cell lung cancer, squamous cell carcinoma of the head and neck, and oligodendroglioma [12–14]. Intratumoral heterogeneity of ^18^F-FDG uptake has been found to be significantly correlated with patient outcomes in sarcoma and cervical cancer [15–17] and has also been used to tailor therapeutic strategies, such as defining the target volume or optimizing the dose distribution in planning for radiotherapy [18–21].

Breast cancer is the most frequently diagnosed solid malignancy in American women and the second most common cause of cancer-related mortality [22]. ^18^F-fluorodeoxyglucose positron emission tomography/computed tomography (^18^F-FDG PET/CT) has been used widely for staging work-ups in breast cancer patients, and several reports have shown that the standardized uptake value (SUV) and metabolic tumor volume (MTV) obtained with the PET/CT scan are useful for predicting survival [23–25]. As far as the authors know, the intratumoral heterogeneity of ^18^F-FDG uptake has not been evaluated or assessed as a prognostic PET parameter in breast cancer.

The objectives of this study were to quantitatively measure the intratumoral metabolic heterogeneity in primary breast cancer on pretreatment ^18^F-FDG PET/CT scans and to assess for any correlation with maximum SUV (SUVmax), MTV, total lesion glycolysis (TLG), disease stage, and prognosis.

## Methods

### Patients

This study was approved by the institutional review board of Kyungpook National University Hospital. A group of 123 patients with invasive ductal carcinoma (IDC) of the breast diagnosed between March 2007 and December 2008 at our hospital were retrospectively evaluated in this study. All patients had undergone ^18^F-FDG PET/CT before treatment. One hundred eighteen patients underwent mastectomy. In 122 patients, systemic chemotherapy and/or hormone therapy were administered. Systemic chemotherapy consisted of doxorubicin (Adriamycin®; Pharmacia & Upjohn, Peapack, NJ, USA) and cyclophosphamide, followed by docetaxel, preoperatively or postoperatively. Hormone therapy was administered to patients with hormone receptor–positive tumors. Patients with human epidermal growth factor receptor 2 (HER2)-positive breast cancer were treated with trastuzumab (Herceptin®, Genentech, South San Francisco CA, USA) for 1 year postoperatively. Postoperative radiation was administered when indicated according to the National Comprehensive Cancer Network (NCCN) guidelines and Recommendations for breast cancer treatment of the Korean Breast Cancer Society. The tumors were classified and staged according to the World Health Organization classification and the TNM staging system [26, 27]. In the patients receiving neoadjuvant chemotherapy, pathologic T and N staging can be influenced by the systemic therapy given before surgical procedures. For those patients, we used the stage assigned before chemotherapy.

### ^18^F-FDG PET/CT acquisition and image analysis

All patients fasted for at least 6 hours, and the blood glucose levels were checked before the administration of ^18^F-FDG. The blood glucose concentration was managed at less than 150 mg/dL in all subjects. Patients with elevated blood glucose levels had their examinations rescheduled. Approximately 8.1 megaBecquerels (MBq) of ^18^F-FDG per kilogram of body weight was injected intravenously, and the patients rested for 1 h before acquisition of the PET/CT images. PET/CT scans were performed using one of two PET/CT systems (Reveal RT-HiREZ®, CTI Molecular Imaging, Knoxville, TN, USA) (Discovery STE®, GE Healthcare, Milwaukee, WI, USA). Following a low-dose CT scan without contrast from the skull vertex to the knee, with the patient in the supine position and breathing quietly, a PET scan with a maximum spatial resolution of 6.5 mm (Reveal PET/CT) or 5.5 mm (Discovery PET/CT) were obtained from the skull vertex to the knees, at 3 min per bed position. The images obtained by the Reveal PET/CT and Discovery PET/CT scanners were reconstructed with a 128 × 128 matrix, an ordered-subset expectation maximum iterative reconstruction algorithm (4 iterations, 8 subsets), a Gaussian filter of 5.0 mm, and a slice thickness of either 3.0 mm (Reveal PET/CT) or 3.27 mm (Discovery PET/CT).

The SUVmax was obtained using the following formula: SUVmax = maximum activity in the region of interest (MBq/gram)/(injected dose [MBq]/body weight [grams]). The MTV was determined as the total number of voxels with threshold SUV of ≥ 40% of the SUVmax in the volume of interest. The TLG was calculated as the MTV multiplied by its SUVmean.

The intratumoral metabolic heterogeneity was represented by the heterogeneity factor (HF) [16], which was determined for each patient as follows: a region of interest (ROI) was manually drawn to fully include the primary tumor and a surrounding region of normal tissue (normal background). The tumor volume was determined with a series of SUV thresholds (e.g., 40%, 50%, 60%, 70%, and 80% of SUVmax) using the semiautomatic software of the workstation (Advantage Workstation 4.3, GE Healthcare). We excluded the values of < 40% from the heterogeneity analysis because a previous study reported that the minimal threshold that represents the actual tumor volume was 40% and the values of < 40% included too much normal tissue background activity [28]. In addition, values of > 80% were also excluded because the volumes were small and the partial volume effect was pronounced [29]. A volume-threshold function of the tumor was acquired by plotting thresholds to volumes. Linear regression analysis was performed and the HF was calculated by finding the derivative (dV/dT) of the volume-threshold function for each tumor. Next, the values of the HF were modified into absolute values so that all resulting values were positive; the more positive the factor, the more heterogeneous the tumor. It took about 1 min to obtain the intratumoral metabolic heterogeneity for each lesion.

### Immunohistochemistry

Immunohistochemical staining was performed on tissue slices from formalin-fixed, paraffin-embedded representative breast tumors. Estrogen receptor (ER), progesterone receptor (PR), and HER2 expression were assessed by immunohistochemistry using commercial monoclonal antibodies for ER (1:200 dilution; Neomarkers, Fremont, CA, USA), PR (1:4,500 dilution; Neomarkers), and HER2 (1:300 dilution; DakoCytomation, Glostrup, Denmark). The iView DAB detection kit (Ventana Medical Systems, Tucson, AZ, USA) was used as the secondary antibody. The results were recorded according to the guidelines of the American Society of Clinical Oncology and the College of American Pathologists [30]. Cases with a HER2 immunohistochemical staining score of more than 2 were tested by HER2 gene amplification using the fluorescence in situ hybridization method. HER2 positivity was defined as an immunohistochemical staining score of 3+ or, in the case of an immunohistochemical staining score of 2+, as positive findings on fluorescence in situ hybridization.

### Statistical analysis

The clinicopathologic factors and survival outcomes data were analyzed for correlation with the HF. Spearman correlation analysis was used to clarify the relations between the PET parameters (HF, SUVmax, MTV, and TLG). Student’s *t*-test was used to compare the differences in the HFs between pairs of groups: T1, T2 combined and T3, T4 combined; N0, N1 combined and N2, N3 combined; M0 and M1; ER-positive and negative; PR-positive and negative; HER2-positive and negative. A one-way analysis of variance (ANOVA) test was used to compare the means of the HFs in each of the T, N, and M stages.

The overall survival (OS) was used as the patient outcome measure. The survival time was measured from the date of the PET/CT scan to the date of death. For survivors, the last follow-up time was used as the endpoint (March 2013). To identify an optimal cut-off for SUVmax, MTV, TLG, and the HF for the prediction of OS, receiver-operating characteristic (ROC) curve analysis was performed. The OS was calculated using the Kaplan–Meier method. The Cox proportional-hazards model was used to assess the potential independence of the effects of the PET parameters on OS after adjustment for the effects of clinicopathologic parameters that significantly affected OS according to the Kaplan-Meier survival analysis.

The MedCalc® statistical package (version 12.3.0.0, MedCalc Software, Mariakerke, Belgium) was used for statistical analysis. A p value of less than 0.05 was considered statistically significant.

## Results

### Patient characteristics

The patient demographic data and tumor characteristics, including the age at diagnosis, the TNM stage, and the hormone receptor status, are shown in Table 1. The mean follow-up for all patients was 53.6 months (range, 7–66 months). Among the patients, 106 (86.2%) were alive (the survival group) and 17 (13.8%) had expired (the death group).

**Table 1:** **Clinical characteristics of patients**

Variable	Number of patients	%
All patients	123	100
Age (years)		
≤45	49	40
>45	74	60
ER status		
Positive	75	61
Negative	48	39
PR status		
Positive	74	60
Negative	49	40
HER2 status		
Positive	41	33
Negative	82	67
T stage		
1	60	49
2	49	40
3	10	8
4	4	3
N stage		
0	63	51
1	29	24
2	18	15
3	13	11
M stage		
0	114	93
1	9	7
Chemotherapy		
No	19	15
Yes	104	85
Hormone receptor targeting therapy		
No	34	28
Yes	89	72
Radiation therapy		
No	50	41
Yes	73	59
Herceptin		
No	102	83
Yes	21	17

ER = Estrogen receptor, PR = Progesterone receptor, HER2 = Human epidermal growth factor receptor 2.

### HF and TNM categories

The distribution of the TNM stages is shown in Table 1. The HFs of T1 and T2 tumors were significantly lower (i.e., less heterogeneous) than those of T3 and T4 tumors (0.18 ± 0.25 vs. 1.60 ± 1.95, p < 0.0001). The HFs of the N0 and N1 tumors were significantly lower than those of the N2 and N3 tumors (0.20 ± 0.27 vs. 0.78 ± 1.49, p = 0.0005). No significant difference in the HFs was found between the patients without (M0) and with (M1) metastases (0.34 ± 0.83 vs. 0.48 ± 0.59, p = 0.6119).

By the ANOVA test, the means of the HFs in each of the T stages, as well as the different N stages, were significantly different (p < 0.001). The test did not correlate significantly with the M stage (Table 2).

**Table 2:** **Results of one-way ANOVA tests, clarifying the differences of the means of the heterogeneity factor (HF) in each of the T, N, and M stages**

Variables	No. of cases (%)	HF (mean)	F-ratio	p value
**T stage**			27.33	< 0.001*
**T1**	60 (48.8)	0.18		
**T2**	49 (39.8)	0.19		
**T3**	10 (8.1)	1.12		
**T4**	4 (3.3)	2.81		
**N stage**			8.07	< 0.001*
**N0**	63 (51.2)	0.19		
**N1**	29 (23.6)	0.22		
**N2**	18 (14.6)	0.40		
**N3**	13 (10.6)	1.30		
**M stage**			0.26	0.612
**M0**	114 (92.7)	0.34		
**M1**	9 (7.3)	0.48		

HF = Heterogeneity factor.

*p < 0.01.

### HF and hormone receptor status/HER2 status

The distribution of the ER and PR status is shown in Table 1. The HF of ER-positive tumors was significantly lower than that of ER-negative tumors (0.22 ± 0.40 vs. 0.54 ± 1.19, p = 0.0319). The HF of the PR-positive tumors was significantly lower than that of the PR-negative tumors (0.23 ± 0.38 vs. 0.53 ± 1.19, p = 0.0475). No significant difference in the HF values was found between the patients with HER2-positive and HER2-negative tumors (0.42 ± 1.05 vs. 0.31 ± 0.67, p = 0.4662).

### HF and other metabolic PET parameters

The SUVmax ranged from 0.9 to 26.3 (mean, 6.8 ± 4.5; median, 5.6). The MTV ranged from 0.55 to 126.00 cm^3^ (mean, 8.73 ± 17.32 cm^3^; median, 3.54 cm^3^). The TLG ranged from 0.0003 to 240.56 (mean, 24.52 ± 43.65; median, 6.84). The HF ranged from 0.02 to 6.72 (mean, 0.35 ± 0.82; median, 0.13).

**Figure 1:**
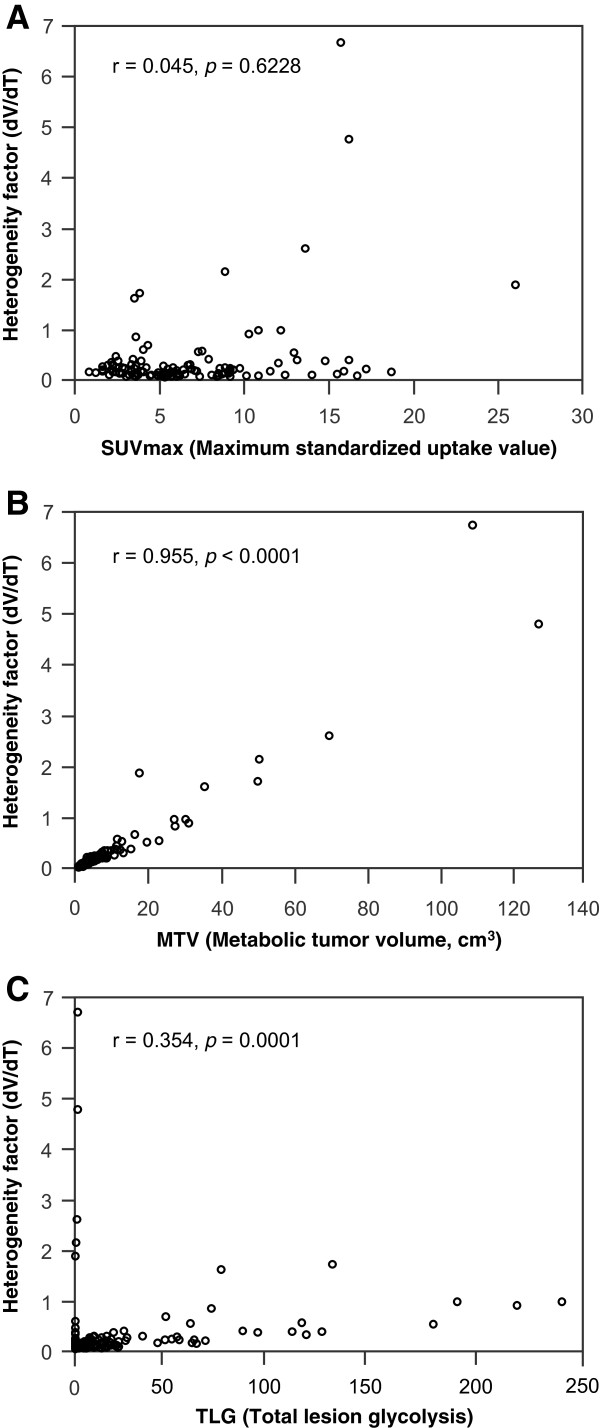
**Correlation between the heterogeneity factor (HF) and metabolic PET parameters. (A)** HF and maximum standardized uptake value (SUVmax), **(B)** HF and metabolic tumor volume (MTV), **(C)** HF and total lesion glycolysis (TLG).

The HF correlated well with the MTV, with a very strong correlation coefficient (r = 0.955; p < 0.0001). Although there was a significant correlation between the HF and the TLG, the correlation coefficient was relatively weak (r = 0.354; p = 0.0001). The HF did not correlate with SUVmax (p = 0.6228) (Table 3, Figure 1).

**Table 3:** **Spearman correlation of the HF with SUVmax, MTV, TLG**

Variables	HF	
	**Spearman’s coefficient of rank correlation (rho)**	**p value**
**SUVmax**	0.045	0.6228
**MTV**	0.955	< 0.0001*
**TLG**	0.354	0.0001*

HF = Heterogeneity factor, SUVmax = Maximum standardized uptake value, MTV = Metabolic tumor volume, TLG = Total lesion glycolysis.

*p < 0.01.

### Survival analysis

The mean HF in the death group was significantly higher than that in the survival group (1.28 ± 1.88 vs. 0.20 ± 0.26, p < 0.0001). The SUVmax and the MTV of the death group were significantly higher than in the survival group (SUVmax: 11.7 ± 5.6 vs. 6.0 ± 3.8, p < 0.0001; MTV: 28.23 ± 38.08 cm^3^ vs. 5.60 ± 7.48 cm^3^, p < 0.0001).

**Figure 2:**
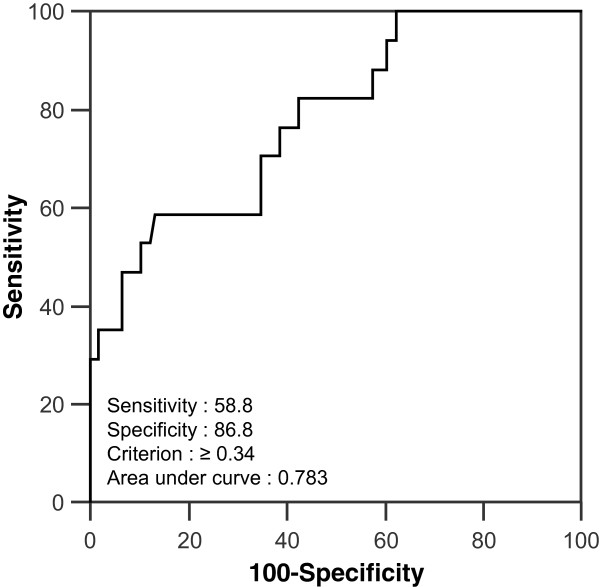
**Receiver operating characteristic (ROC) curve for predicting overall survival in patients with invasive ductal carcinoma of the breast.** Heterogeneity factor (HF): optimal cutoff, 0.34; area under the curve, 0.783; significant error, 0.061.

The ROC curve demonstrated that an HF of 0.34 was the optimal cut-off for predicting the OS (sensitivity, 58.8%; specificity, 86.8%; area under the curve, 0.783; SE, 0.061) (Figure 2). The optimal cut-off values determined using the ROC curve were 5.6 for the SUVmax, 8.55 cm^3^ for the MTV, and 14.43 for TLG. The ROC curve showed no statistical significance for TLG.

**Figure 3:**
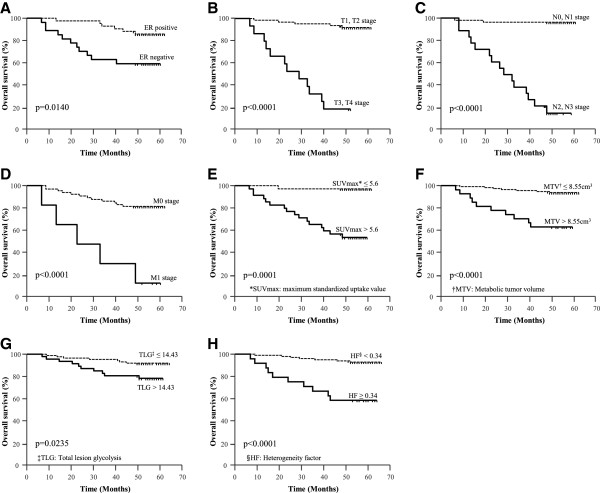
**Kaplan-Meier plots of overall survival (OS) in patients with invasive ductal carcinoma (IDC) of the breast. (A)** Estrogen receptor (ER) status, **(B-D)** TNM stage, **(E)** maximum standardized uptake value (SUVmax), **(F)** metabolic tumor volume (MTV), **(G)** total lesion glycolysis (TLG), and **(H)** heterogeneity factor (HF) significantly affected OS.

A Kaplan-Meier analysis revealed that T stage (1, 2 vs. 3, 4), N stage (0, 1 vs. 2, 3), M stage (0 vs. 1), ER status (+ vs. –), SUVmax (≤ 5.6 vs. > 5.6), MTV (≤ 8.55 cm^3^ vs. > 8.55 cm^3^), TLG (≤ 14.43 vs. > 14.43), and HF (< 0.34 vs. ≥ 0.34) were the significant predictors of OS (Table 4, Figure 3). Age (≤ 45 vs. > 45), PR status (+ vs. –), and HER2 status (+ vs. –) were not predictive of OS. After adjustment for the effects of the clinicopathologic parameters (TNM stage and ER status), the HF and the MTV remained significant predictors of OS. Among the metabolic PET parameters, the best prognostic factor of OS was HF with a cut-off value of 0.34 (Hazard Ratio 3.56; 95% CI 1.3 - 9.9); p = 0.0153) (Table 5).

**Table 4:** **Factors affecting overall survival (OS) in the univariate analysis**

Variables	Total no. of patients	No. of deaths	Median OS (months)	OS		
				p value	Hazard ratio	95% CI for hazard ratio
**Age (years)**						
**≤45**	49	8	59	-	1.00	-
**>45**	74	9	61	0.5251	0.74	0.3 – 1.9
**T stage**						
**T1, T2**	109	5	58	-	1.00	-
**T3, T4**	14	12	28	< 0.0001*	33.03	5.0 – 219.0
**N stage**						
**N0, N1**	63	0	58	-	1.00	-
**N2, N3**	60	17	51	< 0.0001*	27.97	8.7 – 89.6
**M stage**						
**M0**	114	12	57	-	1.00	-
**M1**	9	5	52	< 0.0001*	7.03	0.9 – 55.6
**ER**						
**Positive**	75	6	57	-	1.00	-
**Negative**	48	11	56	**0.0140** ^**†**^	3.24	1.2 – 8.7
**PR**						
**Positive**	74	7	63	-	1.00	-
**Negative**	49	10	57	0.0748	2.34	0.9 – 6.2
**HER2**						
** Negative**	82	11	56	-	1.00	-
**Positive**	41	6	57	0.8154	1.13	0.4 – 3.1
**SUVmax**						
**≤ 5.6**	61	0	57	-	1.00	-
**> 5.6**	62	17	56	0.0001*	18.38	7.1 – 47.7
**MTV (cm** ^**3**^ **)**						
**≤ 8.55**	99	7	57	-	1.00	-
**> 8.55**	24	10	55	< 0.0001*	7.42	2.0 – 27.0
**TLG**						
**≤ 14.43**	80	7	63	-	1.00	-
**> 14.43**	43	10	55	**0.0235** ^**†**^	2.90	1.1 – 8.0
**HF**						
**< 0.34**	99	7	57	-	1.00	-
**≥ 0.34**	24	10	55	< 0.0001*	7.42	2.0 – 27.0

CI = Confidence interval, ER = Estrogen receptor, PR = Progesterone receptor, OS = Overall survival, PR = Progesterone receptor, HER2 = Human epidermal growth factor receptor 2, SUVmax = Maximum standardized uptake value, MTV = Metabolic tumor volume, TLG = Total lesion glycolysis, HF = Heterogeneity factor.

*p < 0.01, ^†^p < 0.05.

**Table 5:** **Multivariate analysis of the metabolic PET parameters after adjusting for the effects of prognostic clinicopathologic parameters (TNM stage and estrogen receptor status)**

Variables	OS		
	p value	Hazard ratio	95% CI for hazard ratio
**SUVmax**			
**≤ 5.6**	-	1.00	-
**> 5.6**	0.1526	4.52	0.6 – 35.4
**MTV (cm** ^**3**^ **)**			
**≤ 8.55**	-		-
**> 8.55**	**0.0183** ^†^	3.45	1.2 – 9.6
**TLG**			
**≤ 14.43**	-	1.00	-
**> 14.43**	0.6103	1.29	0.5 – 3.5
**HF**			
**< 0.34**	-	1.00	-
**≥ 0.34**	**0.0153** ^†^	3.56	1.3 – 9.9

PET = Positron-emission tomography, OS = Overall survival, CI = Confidence interval, SUVmax: = Maximum standardized uptake value, MTV = Metabolic tumor volume, TLG = Total lesion glycolysis, HF = Heterogeneity factor.

^†^p < 0.05.

## Discussion

**Figure 4:**
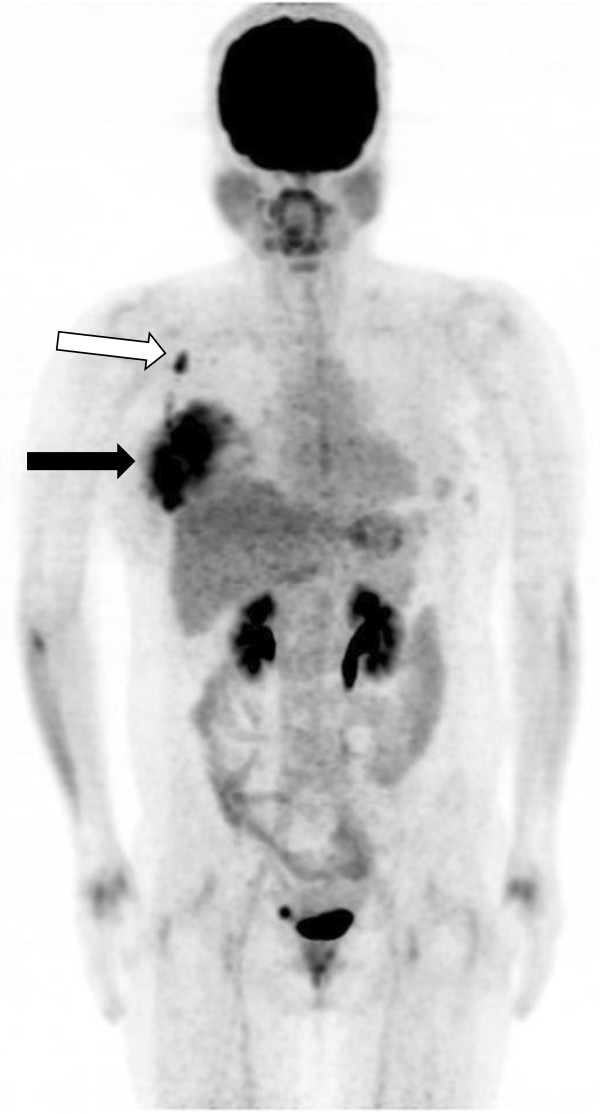
**The maximum intensity projection (MIP) image of an** ^**18**^ **F-FDG PET/CT scan in a patient with invasive ductal carcinoma of the breast and an elevated HF value.** The image shows increased ^18^F-FDG uptake in the right breast (black arrow) and ipsilateral axillary lymph nodes (white arrow). The SUVmax of the right breast mass was 9.0, the MTV was 49.79 cm^3^, the TLG was 276.45, and the heterogeneity factor (HF) was 2.12. This patient died from disease progression 17 months after acquisition of this image.

**Figure 5:**
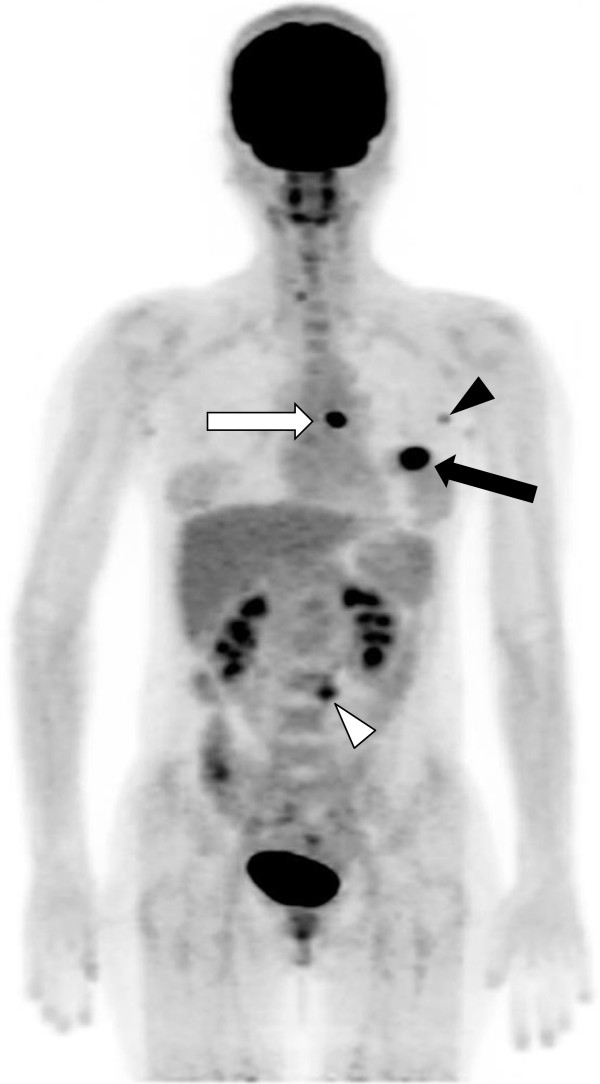
**The maximum intensity projection (MIP) image of an** ^**18**^ **F-FDG PET/CT scan in a patient with invasive ductal carcinoma of the breast and a low HF value.** The image shows focal ^18^F-FDG uptake in the left breast (black arrow), ipsilateral internal mammary (white arrow), and axillary lymph nodes (black arrowhead). There is also focal ^18^F-FDG uptake in an enlarged paraaortic lymph node (white arrowhead). The SUVmax of the left breast mass was 16.9, the MTV was 1.66 cm^3^, the TLG was 21.21, and the heterogeneity factor (HF) was 0.04. This patient was still alive 52 months after acquisition of this image and had no clinical evidence of disease progression.

The tumor microenvironment is inherently heterogeneous and is best characterized as a patchwork of areas of adequate and inadequate oxygen and nutrient delivery, which leads to the epigenetic and genetic adaptations of clones and increases the aggressiveness of the tumor by local invasion and distant metastasis. Tumors undergoing rapid proliferation often outgrow the existing vasculature, creating intratumoral hypoxia [31] and adding complexity to the tumor biology, creating challenges to treatment planning [32–35]. The adaptation of tumors to hypoxia makes them more resistant to treatment, conferring severe resistance to current therapies. ^18^F-fluoromisonidazole (FMISO) has been developed as a PET tracer for hypoxia imaging; a recent report revealed that a ^18^F-FMISO PET/CT scan is an effective imaging tool that can accurately predict therapeutic response by visualizing tumor hypoxia [36] (Figures 4 and 5).

We hypothesized that the metabolic heterogeneity of IDC lesions could be correlated with known prognostic metabolic PET parameters (SUVmax, MTV and TLG), as well as with the TNM stage, and could be an accurate prognostic indicator. According to the results of this study, the MTV was the factor most closely correlated with the HF among the metabolic PET parameters. It was noted that the SUVmax did not correlate with the HF. These findings suggest that intratumoral metabolic heterogeneity, which reflects varying tumoral metabolic characteristics, may correlate more with tumor size than to tumoral glucose metabolic activity. Considering that intratumoral metabolic heterogeneity is associated with heterogeneous tumor hypoxia, previous study results [36], which revealed no correlation between the uptakes of ^18^F-FDG and ^18^F-FMISO, can be similarly understood. The degree of tumoral glucose uptake might not be solely related to hypoxia, which is one of the key characteristics of tumor heterogeneity; other biological factors may be involved.

The results of this study revealed that high intratumoral metabolic heterogeneity was significantly associated with a high T stage and a high MTV. The T stage, determined by the pathologic size and extension of the primary tumor, was strongly associated with the MTV. High intratumoral metabolic heterogeneity in large tumors might be generated by the growth of significant subpopulations of tumor cells that have diverse metabolic characteristics.

We determined that the level of intratumoral metabolic heterogeneity on ^18^F-FDG PET scanning is predictive of prognosis in IDC patients. On multivariate analysis, the results demonstrated that the HF and the MTV were significant prognostic factors for OS, even after adjusting for clinicopathologic prognostic parameters (TNM stage and ER status) that were statistically significant on the univariate analyses. The HF was the superior prognostic factor among the metabolic PET parameters obtained with ^18^F-FDG PET/CT. This measurement could play an important role in designing a personalized treatment plan by providing detailed prognostic information in IDC patients with otherwise identical risk factors. There is limited understanding of the reasons that metabolic heterogeneity of a tumor is linked to survival, but focal hypoxia in a large tumor may be one reason. Cooper *et al.* [37] observed that the regional loss of ER-α expression was consistently present in hypoxic regions of breast cancer tissue. Recent studies that focused on the relationship of HIF-1α tumor expression to the response to chemotherapy or hormone therapy found that increased levels of HIF-1α were associated with resistance [38, 39]. Results of this study revealed that the HF was significantly higher in ER- and PR-negative tumors. These data indicate that heterogeneous hypoxia might be associated with endocrine resistance in breast cancer. The HF in primary breast cancer is evidently associated with the spatial heterogeneity of tumor response to hormone therapy, chemotherapy, and radiation therapy, and has direct implications for overall response to treatment and survival outcomes.

This study has limitations. First, the spatial resolution of PET/CT scanning might affect the accuracy of the HF of small-sized tumors (for example, stage T1 tumors), which could result in an underestimation of heterogeneity. Although we determined that some small-volume tumors showed a high degree of intratumoral metabolic heterogeneity, no one with stage T1 tumors died; therefore we could not perform subgroup analysis with stage T1 breast cancer patients. However, after excluding stage T1 tumors, the results were similar. Further study with a larger cohort would be needed to confirm the value of the HF in smaller sized tumors. Second, the intratumoral metabolic heterogeneity on ^18^F-FDG PET scans can be represented by various methods. The parameter of intratumoral metabolic heterogeneity is not well standardized, although histologic studies have shown good spatial correlation between areas of ^18^F-FDG uptake and histologic findings. A feasible and highly reproducible method for obtaining a heterogeneity parameter representing intratumoral metabolic heterogeneity is warranted. Our future direction is to validate the accuracy of our results in reflecting the true degree of heterogeneity of the tumors, and improve its calculation.

## Conclusions

Among the metabolic parameters obtained with ^18^F-FDG PET scanning, the HF, which had close correlation with the MTV, was the best prognostic factor for predicting the OS in IDC patients. Patients with a high HF value (a more heterogeneous tumor) might be considered candidates for an aggressive neo- or adjuvant therapeutic plan to reduce mortality rates, and should be cautiously followed up.

### Consent

Written informed consent was obtained from the patients for publication of this manuscript and accompanying images.

## Abbreviations

ARG: Autoradiography
ER: Estrogen receptor
F-FDG PET/CT: ^18^F-fluorodeoxyglucose positron-emission tomography/computed tomography
FMISO: Fluoromisonidazole
GLUT: Glucose transporter
HER2: Human epidermal growth factor receptor 2
HF: Heterogeneity factor
HIF-1α: Hypoxia-inducible factor-1α
HK-II: Hexokinase-II
IDC: Invasive ductal carcinoma
MTV: Metabolic tumor volume
OS: Overall survival
PR: Progesterone receptor
ROC: Receiver operating characteristic
ROI: Region of interest
SUV: Standardized uptake value
SUVmax: Maximum standardized uptake value
TLG: Total lesion glycolysis.
